# Stigmatizing attitudes toward Disruptive Mood Dysregulation Disorder (DMDD) in parents vs. non-parents: Effects of medication and genetic etiology

**DOI:** 10.1371/journal.pone.0274185

**Published:** 2022-09-09

**Authors:** Linda M. Isbell, Sungha Kang, Gregory Barysky, Grace Quinn

**Affiliations:** Department of Psychological and Brain Sciences, University of Massachusetts Amherst, Amherst, Massachusetts, United States of America; Lehigh University, UNITED STATES

## Abstract

Stigmatizing attitudes toward children with psychopathology represent a barrier to treatment and well-being, yet almost no research has investigated what contributes to these attitudes. This study examines the effects of medication treatment and genetic etiology on stigmatizing attitudes toward a relatively new and controversial disorder–Disruptive Mood Dysregulation Disorder (DMDD). Participants (159 parents, 225 non-parents) completed a vignette study on Amazon’s Mechanical Turk (MTurk) in which a child displayed behaviors consistent with DMDD. The child was described as either taking psychiatric medication or not, and the vignette described the child’s condition as either genetic or did not mention etiology. Participants who were parents reported greater stigma when the etiology (genetic prime vs. no prime) matched the perceived appropriate treatment (medication vs. no medication). Among parents, a child treated with medication who had a genetic disorder, and a child who was not treated with medication and for whom genetic etiology was not primed, were most stigmatized. No differences emerged among non-parents. These findings highlight the importance of considering multiple factors (parental status, congruence between treatment and perceived disorder etiology) when investigating mental health stigma and underscore the need to further investigate such nuances to inform anti-stigma interventions.

## Introduction

Despite decades of research and efforts to reduce stigma, individuals with psychological disorders continue to be subjected to stigmatizing attitudes and negative stereotypes (e.g., perceived as dangerous, unpredictable [1]). Stigmatizing attitudes toward such individuals exacerbate their suffering beyond the symptoms of psychopathology, hinder treatment approachability, and alienate them from society [2]. Although much of this research has focused on adult psychopathology, the steadily increasing prevalence of childhood psychopathology [3], along with the low rate of treatment utilization for children [4], and the growing number of childhood disorders appearing in the *Diagnostic and Statistical Manual of Mental Disorders* (DSM) [5] all underscore a need to better understand factors that contribute to stigma toward childhood psychopathology.

As an increasing number of children are diagnosed with psychological disorders, concerns about the medicalization of normative childhood behavior (i.e., turning behavioral or emotional problems into medical disorders to be treated) have been raised in the media, by the public, and in academic writing [6, 7]. Advancing the notion that society is turning normal childhood behaviors into medical conditions may fuel stigma toward children diagnosed with and treated for psychological disorders, which may steer affected children and their parents away from necessary treatment.

### Disruptive Mood Dysregulation Disorder (DMDD)

One particularly controversial childhood disorder is Disruptive Mood Dysregulation Disorder (DMDD) [5, 8]. DMDD, a childhood mood disorder recently added to the DSM-5 [5, 9], is characterized by persistent irritability or anger along with severe and recurrent temper tantrums [5, 10, 11], and can be diagnosed in children as young as six. Given that DMDD symptoms can resemble “bad behavior,” it is not surprising that this disorder has been cited as an example of medicalization [8]. As Frances [7] noted, “the idea of turning temper tantrums into a mental disorder is terrible…we should not have the ambition to label as mental disorder every inconvenient or distressing aspect of childhood” (p. 177). Not surprisingly, the diagnostic validity of DMDD remains highly controversial [12].

It is worth noting that the addition of DMDD to the DSM-5 was partly a response to concerns about misdiagnosis of pediatric bipolar disorder and overtreatment of children with antipsychotic medications, which can have potentially serious side effects, and for which the long-term safety is not well-established in children and adolescents [9, 13]. Introducing DMDD in the DSM-5 was expected to address this concern about misdiagnosing pediatric bipolar disorder. Indeed, the rate of DMDD diagnoses has increased while the rate of pediatric bipolar disorder diagnoses has decreased with the DSM-5, but more youth with DMDD diagnoses are prescribed antipsychotic medications [14].

In sum, DMDD is not only likely to invite stigma given perceptions that the disorder reflects the medicalization of “bad” child behaviors, but also because of the frequent use of psychiatric medications for its treatment. Considerable stigma exists around the use of psychiatric medication especially for children and adolescents. For example, results from one nationally representative survey assessing adults’ reports of childhood mental health stigma revealed that a majority of adults believed that stigma resulting from receiving mental health treatment in childhood carries into adulthood, and stigma was especially prevalent for disorders treated with medication [15]. Indeed, the vast majority (86%) in this survey believed that children were being overmedicated for common behavioral problems, and that medicating children would have both immediate and long-term negative effects. Similarly, in another survey with a nationally representative sample, the majority of adults were unwilling to endorse the use of medication (i.e., Prozac) for clinically significant conditions in children [6].

### Biological attributions for psychological disorders

Previous research suggests that mental health stigma can come from multiple sources. For example, believing that the symptoms of psychopathology reflect “bad” behaviors (e.g., temper tantrums, poor self-control, being spoiled) can result in personal attributions that lead people to blame the afflicted (e.g., child) and closely associated others (e.g., parents), resulting in stigma towards both the child and the parents. However, almost all research that has investigated the impact of different attributions for psychological disorders has investigated stigma toward adult patients. During the early phases of this research, scholars theorized that biomedical attributions (e.g., genetics) for mental illness (vs. psychosocial attributions), would reduce stigmatizing attitudes [16] because biologically-based conditions may be more treatable [17]. However, more recent work has provided limited evidence for this claim [18, 19]. Specifically, stigma related to unpredictability, dangerousness, and desire for social distance was not reduced following biological or genetic descriptions of a disorder [1, 19]. In fact, attributions to biological causes for adult psychopathology were associated with greater stigma and desire for social distance [19]. Of note, biological attributions have been found to reduce blame and accountability of individuals with mental disorders [19–22] but this has not resulted in reduced stigma. However, in one set of studies investigating stigma towards childhood ADHD, biological attributions did lead to reductions in social rejection, though these attributions led to greater pessimism about treatability and prognosis, consistent with findings in adult psychopathology [23]. These mixed findings demonstrate that the effects of biological attributions for childhood psychological disorders on stigmatizing attitudes warrant further investigation to identify factors that shape the nuances of such stigma.

### Combined effects of genetic etiology and medication treatment on stigmatizing attitudes

Research generally suggests that stigmatizing attitudes are often greater when psychological disorders are attributed to a biological cause and when psychiatric medication is used for treatment. However, it is possible that stigmatizing attitudes may not simply be a function of *either* genetic etiology or use of psychiatric medication but may depend upon the combination of the two. The aforementioned research suggests that such effects may be additive. That is, a genetic disorder treated with medication may evoke greater stigma than either of these conditions alone, whereas a non-genetic disorder not treated with medication may evoke the least stigma. However, research also suggests an alternative possibility in which the effects of genetic etiology and medication treatment may instead be interactive. That is, stigma may depend on whether perceived disorder etiology is congruent with treatment. The current study explored these possibilities, the latter of which builds upon findings from research in clinical psychology and social cognition, which we describe next.

A growing number of studies reveals that individuals (i.e., clinicians, mental health clients, undergraduate students) often perceive psychological disorders attributed to biological causes to be best treated with medication, and disorders attributed to psychosocial factors to be best treated with non-medication options [24–28]. These findings are consistent with social cognition research demonstrating that individuals believe that causes (e.g., etiology) should match (i.e., be congruent with) effects (e.g., treatment) [29, 30]. Although etiology-treatment congruence (versus incongruence) could result in lower stigma due to expectations for better prognosis, social cognition research suggests an opposite possibility. That is, etiology-treatment congruency may increase the likelihood that perceivers will activate and apply negative stereotypes about individuals with psychological disorders, which would increase stigma.

The idea that etiology-treatment congruence may increase stigma relative to incongruence follows from a significant body of literature in social cognition that demonstrates that when forming an impression of another person, congruent information facilitates categorization, stereotype application, and stereotype-consistent evaluations compared to non-congruent information [31–34]. In other words, given the widespread tendency for people to hold negative attitudes toward individuals with psychological disorders, the more an individual “fits” the stereotype of such a person (e.g., in etiology-treatment congruent conditions), the greater stigma is likely to be. This general notion of “fit” is also supported by a separate line of research demonstrating that individuals’ judgments of the likelihood that a child has a psychological disorder (i.e., conduct disorder, ADHD, panic disorder) are greater when a clinical symptom is paired with consistent contextual information (e.g., deviant peer group, being disliked by friends’ parents) compared to inconsistent information (e.g., non-deviant peer group, being liked by friends’ parents) [35–37]. Taken together, research suggests that perceived congruency between disorder etiology and treatment may result in higher levels of stigma towards childhood psychopathology compared to incongruency.

### Stigma among parents versus non-parents

Investigating childhood psychopathology raises the interesting question of whether parental status may moderate effects on stigmatizing attitudes. Given that parents have considerable first-hand experience with a range of childhood behaviors from observing their own child(ren) and others that they may routinely encounter at schools, playgroups, and in related settings, parents may be particularly attuned to information concerning children and their behaviors. Consistent with this notion, parents have a chronically active parental care motivational system, which facilitates the protection and nurturance of children and influences other social cognitive processes [38]. Although relatively little is known about the ways in which parents’ stigmatizing attitudes may differ from non-parents’, research demonstrates that parents generally hold more socially conservative attitudes, which reflect increased vigilance toward uncertainty and threat, and parents make harsher moral judgments [39, 40]—particularly when their parental role is salient [41] Research also demonstrates that parents perceive greater risks [42] and make more risk averse decisions across a variety of judgmental contexts [43] compared to non-parents. Therefore, based on these findings, we explored the possibility that parents and non-parents may respond differently to a child displaying behavioral problems and symptoms of psychopathology, but we did not generate specific predictions.

### The current study

Taken together, despite consistent evidence that mental health stigma poses significant threats to the prognosis and well-being for adults with psychological disorders [2], little is known about stigma towards childhood psychopathology. DMDD as a new yet controversial diagnosis for children [8, 12] may invite stigmatizing attitudes, given the perception of “bad” child behavior and high rates of medication treatment [8, 9]. However, existing research on the effects of biological attributions and medication treatment on stigmatizing attitudes is somewhat mixed [19, 22, 23], and has mostly focused on adults [19]. The current research aims to enhance the understanding of stigmatizing attitudes toward childhood psychopathology (namely, DMDD) by investigating the combined roles of genetic etiology and the use of psychiatric medication among parents and non-parents. Specifically, we examined whether stigmatizing attitudes may not simply be a function of *either* genetic etiology or use of psychiatric medication, but a combination of these two factors. The first hypothesis is that the effects of genetic etiology and medication may be additive, such that stigma will be greatest in conditions in which medication is used to treat a disorder with a genetic etiology and lowest in conditions in which no medication is used to treat a disorder for which genetic etiology is not primed. A second, alternative hypothesis is that the effects of genetic etiology and medication treatment may instead be interactive, such that stigma depends on perceived etiology-treatment congruence with congruence associated with greater stigma than incongruence. Although we anticipated differences may emerge as a function of parental status, our investigation of how parental status may impact stigma was exploratory. Finally, given the overall exploratory nature of this study, it was not pre-registered.

## Methods

### Participants

Participants were recruited through Amazon’s Mechanical Turk (Mturk) between February and May 2016. The total sample included 530 individuals; however, consistent with much research conducted on Mturk and based on methodological recommendations [44], we excluded individuals who failed two key manipulation and attention check items described below (*n* = 146; 27.5%). Importantly, parents and non-parents were equally likely to be excluded, *χ*^2^(1) = .87, *p* = .35. The final sample included 384 adults aged 18 or older living in the United States. This study was approved by the University of Massachusetts Institutional Review Board, protocol #753; 2016–2891. All participants completed the study on Qualtrics.

### Procedure

After agreeing to the informed consent, participants were randomly assigned to read one of four vignettes in which we manipulated perceived etiology (genetic prime v. no genetic prime) and pharmacological treatment (medication vs. no medication) for a child (Sam, in fourth grade) presenting symptoms consistent with DSM-5’s DMDD [5]. The child’s demographical and symptom descriptions were identical for all participants (e.g., high energy, easily frustrated, mood fluctuations, anger outbursts). Experimental manipulations involved describing his condition as either similar to the genetic disorder that his grandfather and uncle have (i.e., genetic prime) or the disorder that a family friend has (no genetic prime; see bolded text below for manipulation), and either taking psychiatric medication or not (see underlined text below for manipulation). These subtle primes aimed to tap into participants’ implicit stigma [17]. To reduce ambiguity regarding whether the child has a psychological disorder, in all conditions we included that the child’s doctor suspected a mood disorder, and that the child received a psychological evaluation. The full vignette appears below:

Sam is a boy in the 4^th^ grade who goes to Eagle Valley Elementary school. He loves to play soccer with his friends and his favorite class is mathematics. Sam has a lot of energy and sometimes gets easily frustrated when he can’t complete simple tasks. Sam’s parents and teachers have noticed that he has been having more mood fluctuations than usual lately. Sam has been getting into disagreements with his teachers, friends, and family, which tend to end with him walking off in anger. These interactions sometimes unexpectedly escalate to the point where Sam throws books, bangs on his desk at school, or hits the walls at home. Sometimes Sam’s parents don’t know what to do. When they ask him why he gets so angry, Sam says he doesn’t know but that he can’t help it. When Sam is not frustrated or angry, he is like any other kid his age–he enjoys going on bike rides around the neighborhood and playing videogames. At Sam’s annual physical, his doctor said that Sam was in great physical health, but based on his behavior, he suspected that Sam may have a mood disorder. Sam’s parents thought it sounded like it could be similar ***to the genetic disorder that his grandfather and uncle have*** *(****to the disorder that a family friend has****)*. *After a psychological evaluation*, *the doctor prescribed medication to help stabilize Sam’s mood*. *Now Sam takes this medication every day after breakfast*. *(After a psychological evaluation*, *the doctor did not prescribe medication*).

After reading the vignette, participants completed several questionnaires, which included key questions to assess the extent to which participants endorsed genetic and non-genetic causes for the child’s condition. Participants were asked, “In your opinion, how likely is it that Sam’s situation might be caused by ….” (1) “stressful circumstances in his life”, (2) “the normal ups and downs of life”, and (3) “a genetic or inherited problem.” Participants reported their responses along a scale from 1 (*very unlikely)* to 6 *(very likely)*. Participants also reported how serious they considered Sam’s problems, if any, to be by selecting one of the following options: *not at all serious*, *not very serious*, *somewhat serious*, and *very serious*.

To assess stigma, participants indicated their agreement with a series of nine statements using a scale from 1 (*strongly disagree*) to 6 (*strongly agree*). These statements included: (1) If Sam let people know he was in treatment, he would lose some of his friends, (2) Being around Sam would make me feel uncomfortable, (3) Children like Sam are unpredictable, (4) Sam should feel embarrassed about his behavior, (5) Sam’s parents should feel embarrassed about Sam’s behavior, (6) If Sam’s parents let people know that Sam was in treatment, they would lose some of their friends, (7) Sam should feel afraid to tell others about his situation, (8) Sam’s parents should feel afraid to tell others about his situation, and (9) Members of Sam’s family would be better off if Sam’s situation was kept secret.

Next, participants completed seven multiple choice questions to assess attention to the vignette they read. Embedded in these were two critical attention/manipulation check questions that assessed whether participants attended to two key pieces of information: whether the child’s difficulties were similar to his grandfather’s and uncle’s, or a family friend’s, and whether the child’s doctor prescribed medication or not. Individuals who incorrectly responded to these attention and manipulation check questions were excluded from analyses.

To explore possible differences in beliefs about medicalization (i.e., turning behavioral or emotional problems into medical disorders to be treated) as a function of parental status, we asked participants to report the extent to which they agreed or disagreed with a key statement from the 2002 National Stigma Study-Children [15] that specifically assesses beliefs about medicalization: “Doctors today are overmedicating children with common behavior problems.” Participants responded using a scale from 1 (*strongly disagree*) to 6 (*strongly agree*). Participants also responded to the other four items from this survey, which measure general attitudes toward using psychiatric medications in children and assess beliefs about the consequences of such medication-use (e.g., “Medications for children with behavior problems turn kids into ‘zombies’”). These items are not directly related to medicalization, and for this reason, they appear in the S1 Appendix.

After completing all questionnaires, participants provided demographic information and were debriefed.

## Results

### Participant characteristics

The majority of participants identified as female (*n* = 215; 56%) with 167 identifying as male (43.5%), and two as other (0.5%); on average, they were 36.93 years old (*SD* = 12.89). Participants were 81.5% White (*n* = 313), 6.8% Black (*n* = 26), 7.6% Asian or Pacific Islander (*n* = 29), 0.5% Native American (*n* = 2), and 3.6% Other (mostly biracial or mixed race; *n* = 14). Participants’ median income fell in the $40,000 to $50,000 range. One hundred fifty-nine participants (41.4%) identified as parents and 225 (58.6%) identified as non-parents. There were significantly more women than men in the parent sample (female *n* = 110; male *n* = 49), *χ*^2^(2) = 19.87, *p* < .001, though the non-parent sample had a more equal gender distribution (female *n* = 105; male *n* = 118; other = 2). There were no significant differences in race/ethnicity distribution across parents and non-parents, *χ*^2^(4) = 8.43, *p* = .08.

### Attitudes toward psychiatric medication use in children

Overall, the vast majority of participants (*n* = 304; 79.17%) agreed with the statement that doctors are over-medicating children with common behavioral problems–reflecting a belief in medicalization. An analysis of participants’ responses to this item as a function of participants’ parental status revealed that parents (*M* = 4.52; *SD* = 1.12) agreed more strongly with this statement than did non-parents (*M* = 4.24; *SD* = 1.28), *t*(382) = 2.24, *p* = .025; Cohen’s *d* = 0.23. This finding is consistent with our expectation that parents and non-parents may respond differently in our experimental vignette task. For this reason, participants’ parental status is included as an independent variable in our analyses (For analyses of the other four items from the 2002 National Stigma Study-Children [15], see Table 1 in S1 Appendix).

### Manipulation check: Effects of the genetic prime on attributions

The extent to which participants agreed that the child’s condition might be caused by a genetic problem was analyzed as a function of the three independent variables (genetic prime, medication, and parental status) using ANOVA. Only a main effect of genetic prime emerged, revealing that individuals who received the prime judged the child’s disorder as more likely to be due to genetics (*M* = 4.33; *SD* = 1.15) compared to those who did not receive the prime (*M* = 3.94; *SD* = 1.08), *t*(1,380) = 3.42, *p* < .001, Cohen’s *d* = .35; all other *p*s > .12.

Next, we averaged the two items tapping non-genetic causes for the child’s condition (stress, normal ups and downs in life; *r* = .39, *p* < .001) and conducted a similar analysis, which revealed only a main effect of genetic prime, all other *p*s > .099. Specifically, those who received the genetic prime judged non-genetic causes to be less likely to explain the child’s condition (*M* = 3.78; *SD* = .98) compared to those who did not receive the prime (*M* = 3.98; *SD* = .90, *t*(381) = 1.96, *p* = .05, Cohen’s *d* = .20.

Taken together, these results demonstrate that the genetic primes influenced perceptions of disorder causality as expected.

### Stigma as a function of genetic prime and medication treatment

To assess stigmatizing attitudes toward the child described in the vignette, we formed a composite measure by computing the mean agreement on the nine stigma questions (α = .81) for each participant. This stigma measure was then analyzed in a 2 (genetic prime v. no genetic prime) x 2 (medication v. no medication) x 2 (parent v. non-parent) between-subjects analysis of variance (ANOVA). Parents and non-parents reported similar levels of stigma overall (parents: *M* = 2.80; *SD* = 0.75; non-parents: *M* = 2.84; *SD* = 0.77), *F*(1, 382) = 0.37, *p* = .54. However, a three-way interaction between all independent variables revealed differences as a function of parental status, *F*(1, 376) = 4.25, *p* = .04, η^2^ = .01 (see *Table 1*).

**Table 1:** Average composite stigma by etiology and treatment conditions.

	Parents		Non-Parents	
	*n*	Mean (*SD*)	*n*	Mean (*SD*)
Genetic Prime				
Medication	56	2.91 (0.80)	48	2.74 (0.78)
No Medication	43	2.68 (0.65)	73	2.86 (0.78)
No Genetic Prime				
Medication	34	2.64 (0.72)	44	2.91 (0.85)
No Medication	26	2.93 (0.81)	60	2.86 (0.68)

**Fig 1:**
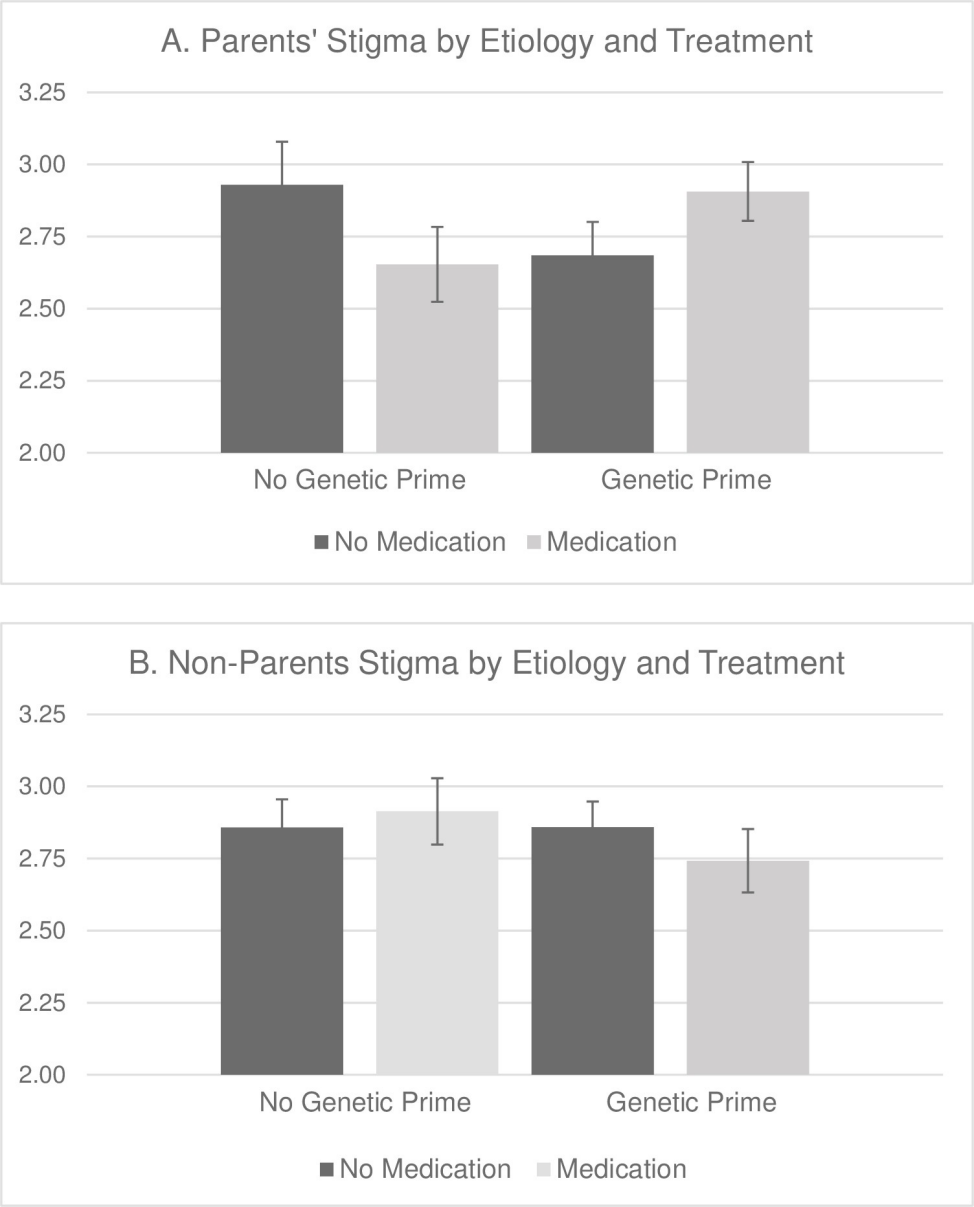
Stigmatizing Attitudes as a Function of Etiology and Treatment for Parents (A) and Non-Parents (B).

To investigate the three-way interaction and examine differences among parents and non-parents, we conducted 2 (genetic prime v. no genetic prime) x 2 (medication v. no medication) ANOVAs for parents and non-parents separately. For parents, a two-way interaction emerged between genetic prime and treatment, *F*(1, 155) = 4.08, *p* = .045, η^2^ = .03, as shown in *Fig 1A*. This interaction reveals that parents reported greater stigma when the cause of the child’s condition matched what is perceived to be the appropriate treatment. Congruent conditions (i.e., medication used in the genetic prime condition and no medications used in the no genetic prime condition) did not significantly differ from one another, *t*(155) = .13, *p* > .50. Likewise, non-congruent conditions (i.e., no medication used in the genetic prime condition and medication used in the no genetic prime condition) did not significantly differ from one another, *t*(155) = .18, *p* > .50; however, the congruent and non-congruent conditions were significantly different, as reflected by the significant interaction. Because there were significantly more women than men in our parent sample, follow-up analyses were conducted to determine whether the congruence effect varies by parent gender. We found no gender differences in stigma as a function of perceived congruence among parents; mothers (*M* = 2.86; *SD* = 0.77) and fathers (*M* = 3.05; *SD* = 0.87) similarly endorsed greater stigma in the congruent conditions compared to incongruent conditions (mothers: *M* = 2.64, *SD* = 0.66; fathers: *M* = 2.74; *SD* = 0.73), *F*(1, 155) = 0.11, *p* = .75, η^2^ = .001. Further, the congruency effect remained significant when gender was included in the analysis, *F*(1,155) = 4.35, *p* = .04, η^2^ = .027.

Among non-parents, the two-way interaction between genetic prime and medication treatment was not significant, *F*(1, 221) = 0.68, *p* = .41, η^2^ = .003, nor was the main effect of genetic prime, *F*(1, 221) = 0.66, *p* = .42, η^2^ = .003, or the main effect of medication treatment, *F*(1, 221) = 0.08, *p* = .77, η^2^ = .000, indicating that stigma did not vary as a function of genetic prime or medication treatment (see *Fig 1B*). Including gender in this analysis did not alter these findings, all *p* > .25.

### Supplementary analysis: Perceived seriousness of the child’s problems

We conducted supplementary analyses to explore the possibility that differences in perceived seriousness of the child’s problems may shed additional light on our findings. We first explored whether there are differences in seriousness ratings between parents and non-parents, and then explored whether there are differences in seriousness ratings between etiology-treatment congruent and incongruent conditions for parents and non-parents separately. Given the ordinal nature of responses to the perceived seriousness question, we used the Mann-Whitney U test [45], which tests the null hypothesis that for two randomly drawn observations from separate groups (i.e., a seriousness rating from a group of parents and from a group of non-parents), the probability of the observation from the first group being higher than the observation from the second is equal to the probability of the second being higher than the first.

First, examining seriousness ratings for parents and non-parents, we found that ratings from parents were significantly higher than from non-parents (mean rank 210.05 versus 180.10), *U* = 15,097.50, *z* = -3.03, *p* = .002 (See Fig 1 in S1 Appendix for response distribution). This demonstrates that parents tended to perceive the child’s behavior as more serious than non-parents.

Next, we examined seriousness ratings for etiology-treatment congruent and incongruent conditions for parents and non-parents separately. For parents, we found a trend in which ratings in congruent conditions were non-significantly higher than in incongruent conditions (mean rank 85.80 versus 73.82), *U* = 3,633, *z* = 1.864, *p* = .062 (See Fig 2A in S1 Appendix for response distribution). Thus, parents showed a slight tendency to perceive the child’s behavior as more serious in congruent compared to incongruent conditions. For non-parents, we found no difference between congruent and incongruent conditions (mean rank 113.44 versus 112.59), *U* = 6,366, *z* = .117, *p* = .907 (See Fig 2B in S1 Appendix). Together these findings suggest that the differences in stigma that emerged among parents may be driven at least in part by differences in perceived seriousness of the child’s problems.

## Discussion

This study examined stigmatizing attitudes toward DMDD–a new and controversial DSM childhood disorder [5, 8]–as a function of etiology (genetic prime v. no genetic prime), treatment (medication v. no medication), and participant parental status (parent v. non-parent). Parents reported greater stigma when disorder etiology was congruent with treatment. These findings build on research demonstrating etiology-treatment congruence effects in perceptions of treatment appropriateness [24, 25] and provide new evidence that stigmatizing attitudes are greater in these conditions–specifically for parents. In addition, these findings follow from social cognition research [31–34] that demonstrates that categorization, stereotype application, and stereotype-consistent evaluation is facilitated when information is congruent relative to non-congruent. That is, given the widespread tendency for the public to hold negative attitudes toward those with psychological disorders, the more an individual “fits” the stereotype of a someone with a psychological disorder, the greater stigma is likely to be.

Some of our data suggest that congruence was associated with slightly greater perceptions that the child in our vignette had more serious problems, which may account for the higher level of stigma. However, our data are unable to provide definitive evidence for the specific mediator(s) of our effects. Other possibilities exist, including that the child in the congruent conditions may have been perceived as more dangerous than the child in the incongruent conditions, or may have triggered prognostic pessimism. Such perceptions also tend to increase stigma [19]. Future research is needed to investigate these possibilities. Identifying the key mediator(s) will be critical for informing efforts to design effective interventions that target specific mechanisms that drive stigma.

Our decision to experimentally manipulate medication (versus no medication) treatment was motivated by our interest in the effects of medication-use versus non-use specifically given that this variable is directly related to medicalization, as well as prior research on the relation between biological attributions, medication-use, and stigma. Although many clinical disorders can be effectively treated with medication and/or other non-pharmacological treatments (e.g., psychotherapy), our study did not explore these alternatives. Instead, we built on research demonstrating that individuals believe that medication is a more appropriate and effective treatment for disorders that are believed to be biologically caused (e.g., genetic) compared to disorders that are not [19, 24–28]. Indeed, this belief is heavily reinforced in advertising for psychiatric medications, which widely promotes the notion that psychological disorders have a biological etiology and that medications are the appropriate treatment [19]. It is worth noting however, that a small number of studies have found that biological attributions for depression have been associated with preferences for non-pharmacological treatments (e.g., psychotherapy) and resulted in reduced stigma compared to pharmacological treatments [46]. Additionally, in one study, biopsychosocial attributions for depression presented as malleable (i.e., influenced by environmental factors) resulted in greater preferences for psychotherapy than medication compared to conditions in which only a biological attribution was presented [47].

Finally, it is important to note that in our no genetic prime condition, we did not specifically indicate the cause of the disorder, but instead used a manipulation to reduce the likelihood that a genetic cause would be inferred in comparison to the genetic prime condition. As our results demonstrate, our manipulation successfully achieved this. Nonetheless, future work is needed to extend this research to investigate treatments beyond medication and to a variety of conditions in which different disorder etiologies are specified. This approach will likely yield stronger effects than those we found using relatively subtle experimental manipulations.

Taken together, our findings highlight that the links between disorder etiology, treatment, and stigma may be more nuanced than previously thought, and therefore warrant further investigation. Indeed, the effects of biological attributions for mental illnesses can vary depending on how stigma is conceptualized and investigated, and can result in increased desire for social distance, perceptions of unpredictability and dangerousness, and prognostic pessimism, but decreased blame [19–22]. The current findings suggest that some of these effects may also emerge when investigating different etiology-treatment combinations (congruent versus incongruent)–moving research beyond investigations of biological attributions for mental illness. This novel hypothesis should be further explored in both child and adult psychopathology research.

### Parental status and stigma

The effects we uncovered in our experiment are specific to parents, and no effects emerged among non-parents. This finding suggests that parents may be more sensitive than non-parents to information concerning DMDD, and perhaps to childhood psychopathology in general. Overall, parents did perceive the child’s problems in our vignette to be more serious than non-parents, which is in line with research highlighting differences in social cognition among parents and non-parents [38]. Such differences may be rooted in different motivational systems in parents that can trigger greater vigilance and attention to details particularly in response to information about children [38]. Parents may have also been more attuned to different amounts of risk in our experimental conditions, compared to non-parents [42, 43]. Additionally, our finding that parents showed greater endorsement of the belief that children are being overmedicated for common behavioral problems is consistent with research demonstrating that parents tend to make harsher moral judgments than non-parents [39, 41].

Our findings contrast with those reported by Pescosolido and colleagues’ [15], who found that stigmatizing attitudes and beliefs toward childhood mental illness were similar regardless of parental status. Importantly however, these studies are methodologically distinct, which may contribute to the divergent conclusions. For example, Pescosolido et al. [15] obtained results from a nationally representative sample in response to a survey that focused on mental illness in general (rather than a specific illness or clinical presentation), whereas we assessed attitudes in response to a vignette in which a child displayed symptoms consistent with DMDD. Of note, however, our findings regarding the high rate of stigmatized attitudes against the use of psychiatric medications in children is comparable to Pescosolido et al.’s [15] findings.

The difference in our findings and those of Pescosolido et al. [15] underscores the need for additional research investigating the role of parental status on stigmatizing attitudes. Although our study is unable to provide a definitive explanation for why differences emerged as a function of parental status, parents were more sensitive than non-parents to differences in disorder etiology and treatment, which suggests that efforts to reduce stigma may require slightly different messages or approaches for parents and non-parents. For example, compared to non-parents, parents may require more detailed, refined, and contextualized messages than non-parents. Further, our finding that parents more strongly endorsed the belief that children are being overmedicated for common behavioral problems suggests that it may be necessary to target different beliefs that parents and non-parents may have regarding childhood behaviors and psychopathology. For example, parents may be more likely than non-parents to believe in medicalization–a belief that may need to be specifically targeted in anti-stigma interventions for parents. Finally, future research should consider that general differences in exposure to children and “expertise” with childhood behaviors may underlie our effects. In this case, experience and exposure to children may be a more general moderator than parental status. If so, teachers, for example, may show similar effects regardless of whether they are parents.

### Limitations

This study has several limitations. First, as noted earlier, we did not explore the effects of different treatments (e.g., psychotherapy) for DMDD or various causes (e.g., psychosocial). Causes and treatments for mental disorders are the product of many factors, are interrelated, and are more complex than we captured in our study. Future research should build on the current work by crafting more detailed presentations and conditions. It is worth noting however, that narrative descriptions of the type we provided are very common in day-to-day conversations, highlighting a strength of our work. Nonetheless, the use of vignettes may limit generalizability. Participants responded to hypothetical situations; attitudes toward a real child may differ from those elicited by vignettes, particularly if rich behavioral observations are available. Our results likely underestimate stigma experienced in real-world contexts. Given that much research on stigmatizing attitudes relies on vignettes and surveys, research is needed that can capture these attitudes and associated behaviors in more real-world settings. Relatedly, our findings may not generalize to parents with a child diagnosed with DMDD or other mental health conditions. Additionally, participants were drawn from an online survey platform, which provided a relatively diverse sample, but may have led to reduced engagement with the vignettes compared to alternative methods, though we employed rigorous manipulation and attention check measures.

Finally, although our stigma scale showed very good internal consistency demonstrating that the scale is reliable, our items may tap multiple facets of stigma. However, this limitation is not unique to our work—a recent review [48] identified over 400 published measures of mental illness stigma that have been used since 2004–67% of which were created for specific studies–and found that many measures assess multiple components or types of stigma in a single scale. This is a more general and significant limitation in the stigma literature, which appears to reflect differences in how researchers conceptualize stigma. Moving forward, the development of a unified conceptualization of stigma is needed along with the use of well-validated scales so that findings can be meaningfully compared across studies.

### Future directions and conclusions

The current findings highlight important questions and opportunities for future research. Research investigating stigma associated with childhood psychopathology is alarmingly scarce, despite that diagnoses of childhood psychopathology and the number of childhood mental disorders in the DSM-5 have increased. What little work has been done suggests that children, like adults, are subject to considerable stigma. Evidence-based stigma-reduction interventions are urgently needed. To accomplish this, it is essential to first develop a comprehensive understanding of the factors that drive stigma to ensure that these interventions are well-informed by research [18]. This is particularly important given that many past attempts to reduce stigma have not been successful [18]. For example, early efforts that “blamed the brain” for psychological disorders were predicated on the assumption that biological attributions would reduce stigma; however, these efforts have frequently failed and sometimes even backfired resulting in increased stigma [18]. Intervention efforts (e.g., anti-stigma public service announcements, other educational campaigns) will benefit from a deeper understanding of factors that contribute to stigma to ensure more reliably positive outcomes.

In future work, it will be important to consider accumulating evidence that stigma associated with specific psychological disorders (e.g., ADHD, depression, DMDD) can differ [2, 23, 49]. This is not surprising given how different various psychological disorders are from one another. Depending on the specific factors that contribute to stigma for specific disorders, different anti-stigma messages and interventions may be needed [17–19, 49]. Further, anti-stigma efforts and interventions also will likely need to be tailored to different recipients. For example, as suggested earlier, parents and non-parents may benefit from messages that emphasizes different types of information about childhood disorders, and parents who have a child who is affected by psychopathology may need even more carefully tailored messages to reduce stigma and encourage help seeking [50]. Such possibilities are very important and promising avenues for future inquiry.

In conclusion, given the well-documented adverse effects of stigma on adults with psychopathology, it is essential to develop a deeper understanding of stigma toward childhood psychopathology and the factors that contribute to it. Because early identification and treatment is crucial in better prognosis of childhood psychopathology, reducing stigma is not only a high priority for the wellness of affected children and families, but also because failure to treat childhood psychopathology poses a great societal cost [17]. The current research contributes to the growing body of knowledge that aims to reduce this cost to children, families, and society.

## Supporting information

S1 Appendix Supplementary analyses. (PDF)
