# Supplementary material for: Stigmatizing attitudes toward Disruptive Mood Dysregulation Disorder (DMDD) in parents vs. non-parents: Effects of medication and genetic etiology
Source: PLoS One. 2022 Sep 9;17(9):e0274185. doi: 10.1371/journal.pone.0274185 (PMC9462715; doi:10.1371/journal.pone.0274185)

Stigmatizing attitudes toward Disruptive Mood Dysregulation Disorder (DMDD) in parents vs. non-parents: Effects of medication and genetic etiology

Linda M. Isbell*, Sungha Kang, Gregory Barysky, and Grace Quinn

---

S1 APPENDIX. SUPPLEMENTARY ANALYSES.

Table 1. Agreement with 2002 National Stigma Study-Children Statements as a Function of Parental Status

Participants' agreement with all five statements from the 2002 National Stigma Study-Children [15] appear in Table 1 as a function of parental status. These statements tap into beliefs that individuals may have about using psychiatric medications in children. Notably, only the first item ("Doctors today are overmedicating children with common behavior problems") specifically assesses beliefs about medicalization, as described in the main text. The other items focus more specifically on the consequences of using psychiatric medications for children. As reported in the text, parents showed significantly greater endorsement of medicalization (item 1) than did non-parents, $t(382) = 2.24$, $p = .025$; Cohen's d = 0.23. No effects emerged for any of the other items, all $p > .22$. The means and standard deviations for all items appear in Table 1.

Table 1

	Parents (n=159)	Non-Parents (n=225)	
1. Doctors today are over-medicating children with common behavior problems.	4.52 (1.12)	4.24 (1.28)	$t(382) = 2.24$, $p = .025$; Cohen's d = .23
2. Giving medications to children with behavior problems will have long-term negative effects on their development.	3.86 (1.25)	3.73 (1.36)	$t(382) = .90$, $p = .37$; Cohen's d=.09
3. Giving children psychiatric medications when they are young only puts off dealing with their real problems.	3.72 (1.42)	3.54 (1.45)	$t(382) = 1.22$, $p = .23$; Cohen's d = .12
4. Medications for children with behavior problems turn kids into "zombies."	3.45 (1.32)	3.32 (1.34)	$t(382) = .99$, $p = .32$; Cohen's d = .10
5.Medications for behavior problems just prevent families from working out problems themselves.	3.26 (1.46)	3.23 (1.39)	$t(381) = .22$, $p = .82$; Cohen's d = .02

Figure 1. Distribution of Responses to "How serious would you consider Sam's problems, if any, to be?" for Parents and Non-Parents

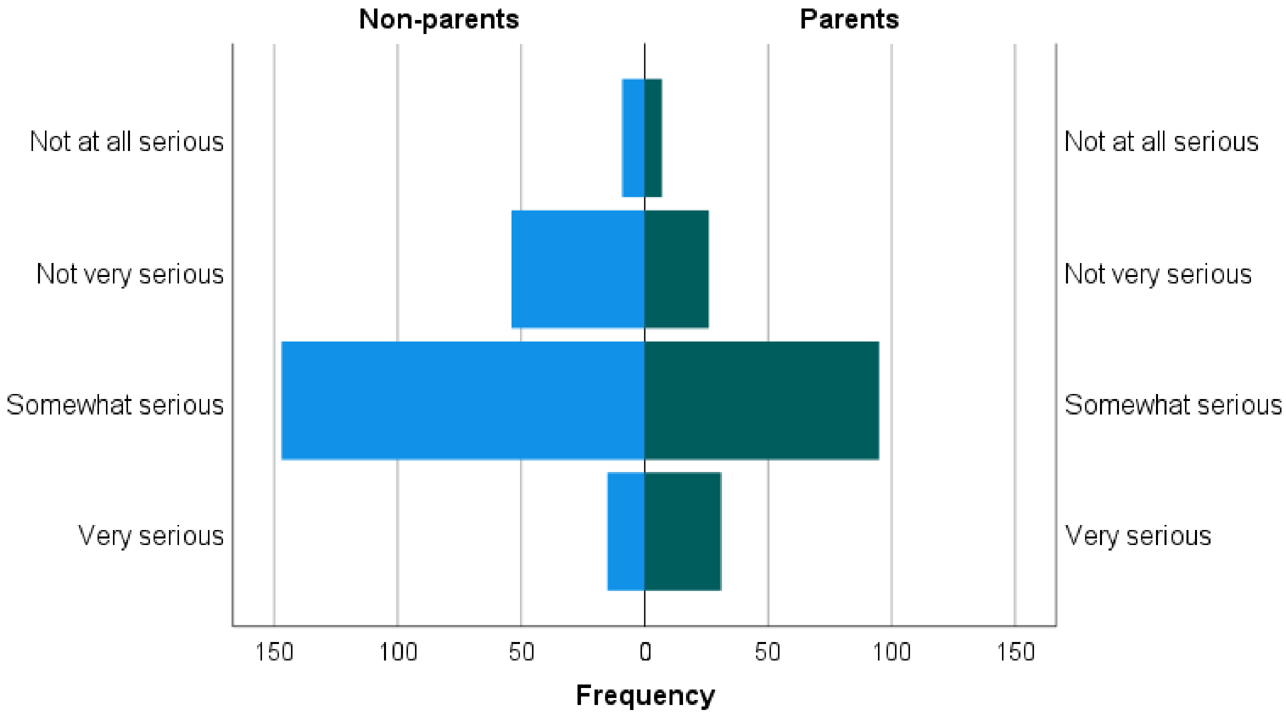

Figure 2: Distribution of Responses to "How serious would you consider Sam's problems, if any, to be?" for Etiology-Treatment Congruent and Incongruent Conditions for Parents (A) and Non-parents (B)

A. Parents

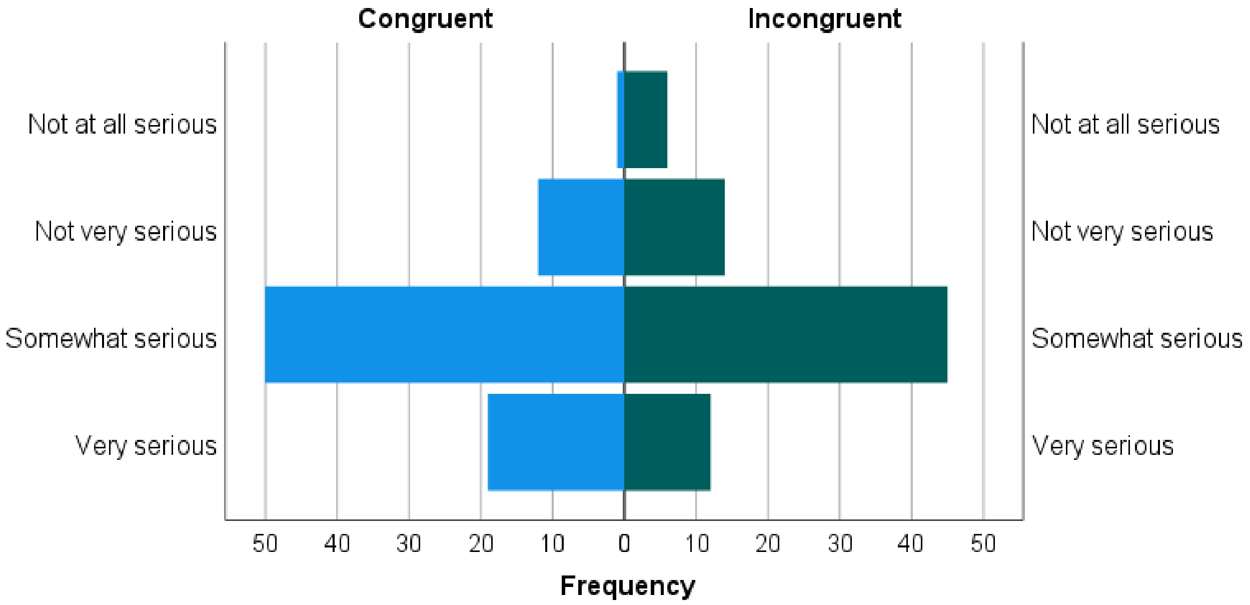

B. Non-parents

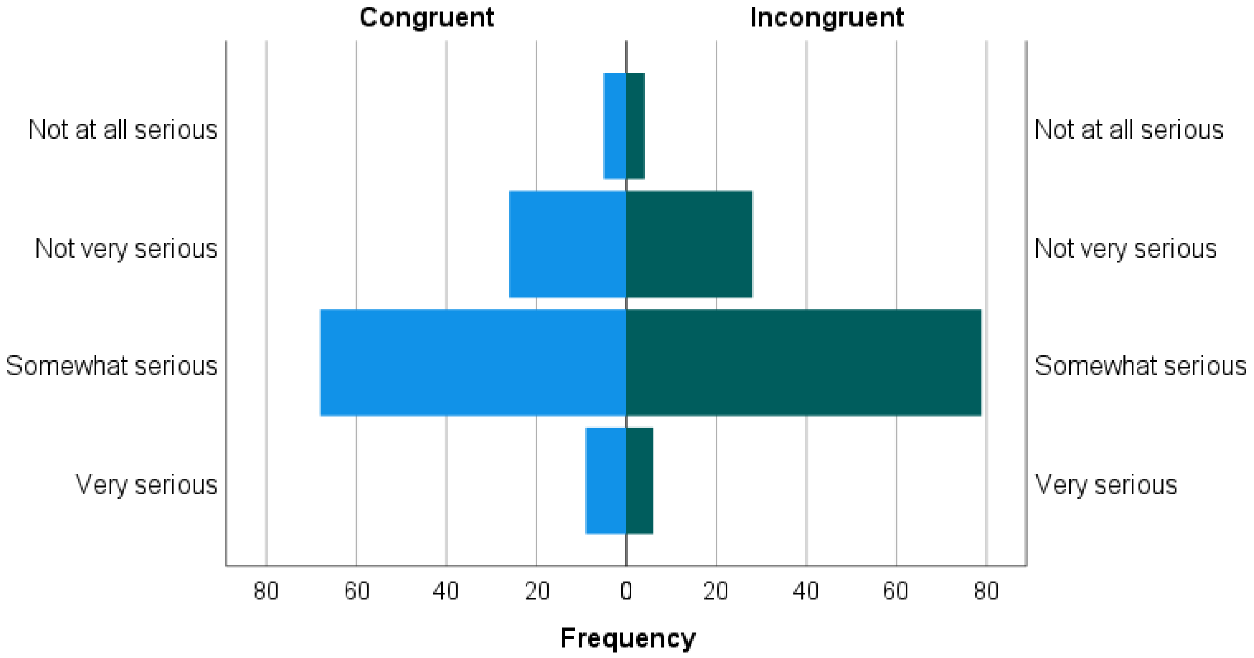

Supplement: S1 Appendix — (PDF) [file pone.0274185.s001.pdf]
